# Chronic Moderate Alcohol Intakes Accelerate SR-B1 Mediated Reverse Cholesterol Transport

**DOI:** 10.1038/srep33032

**Published:** 2016-09-13

**Authors:** Menghua Li, Yan Diao, Ying Liu, Hui Huang, Yanze Li, Peizhu Tan, Huan Liang, Qi He, Junhui Nie, Xingli Dong, Yang Wang, Lingyun Zhou, Xu Gao

**Affiliations:** 1 Department of Biochemistry and Molecular Biology, Harbin Medical University, Harbin, China; 2 Translational Medicine Center of Northern China, Harbin, China; 3 Patent Examination Cooperation Center of the Patent Office, SIPO, Sichuan, China; 4 Department of Gastroenterology, Heilongjiang Province Hospital, Harbin, China; 5 Department of Clinical Laboratory, Harbin Medical University Cancer Hospital, Harbin, China

## Abstract

Cholesterol is essential for all animal life. However, a high level of cholesterol in the body is strongly associated with the progression of various severe diseases. In our study, the potential involvement of alcohol in the regulation of high density lipoprotein (HDL) receptor scavenger receptor class B and type I (SR-B1)-mediated reverse cholesterol transport was investigated. We separated male C57BL/6 mice into four diets: control, alcohol, Control + HC and alcohol + HC. The SR-B1 level and 1,1′-dioctadecyl-3,3,3′,3′-tetramethylindocarbocyanine perchlorate- high- density lipoprotein (DiI-HDL) uptake were also measured in AML12 cells and HL7702 cells treated with alcohol. The control + HC diet led to increased hepatic triglyceride and cholesterol levels while alcohol + HC led no significant change. Compared with that of the control group, the SR-B1 mRNA level was elevated by 27.1% (P < 0.05), 123.8% (P < 0.001) and 343.6% (P < 0.001) in the alcohol, control + HC and alcohol + HC groups, respectively. In AML12 and HL7702 cells, SR-B1 level and DiI-HDL uptake were repressed by SR-B1 siRNA or GW9662. However, these effects were reversed through alcohol treatment. These data suggest that a moderate amount of alcohol plays a novel role in reverse cholesterol transport, mainly mediated by PPARγ and SR-B1.

Recently, the pros and cons of drinking has become a focused issue. As reported, 4% of the world’s morbidity is due to inappropriate drinking^1^. Excessive drinking can lead to more than 60 types of medical problems, including mouth, esophagus, throat, breast and liver cancer; neurological systemic diseases; cirrhosis; and alcohol addiction^2^,^3^. However, most alcohol-associated diseases occur in a dose-dependent manner. Moderate alcohol drinking can reduce the risk of many diseases^4^,^5^: the generation and development of insulin resistance and type 2 diabetes^6^,^7^, dementia, cognitive disorders, osteoporosis and cancer^8^,^9^,^10^. In addition, moderate drinking can also prevent the occurrence of major cardiovascular disease^11^,^12^. Long-term moderate drinking can increase blood HDL-C and reduce LDL-C^13^.

Cardiovascular disease is one of the leading cause of morbidity and mortality in developing nations. Myocardial infarction and stroke as risk factors of cardiovascular disease are mainly caused by atherosclerosis. Atherosclerosis is caused by chronic fat, especially cholesterol deposition in the arterial wall. Cholesterol is required to maintain both membrane structural integrity and fluidity. Cholesterol also serves as a precursor for the biosynthesis of steroid hormones, bile acids and vitamin D^14^. However, excess cholesterol in the body will lead not only to atherosclerosis but also to a number of diseases, including gallstones^15^, osteoporosis^16^, obesity^17^, etc. Cytochrome P450 7A1 (CYP7A1) is the first rate-limiting enzyme that coverts cholesterol into bile acids. Meanwhile, numerous clinical trials have shown a relationship between serum high-density lipoprotein and cardiovascular disease^18^. The protective effects of HDL cholesterol may occur through participation in reverse cholesterol transport (RCT), which is a multi-step process resulting in the net movement of cholesterol from peripheral tissues back to the liver via the plasma^19^. Three primary liver surface receptors are involved in this process: LDLR^20^, LRP1^21^,^22^ and SR-B1^23^. SR-B1 as an HDL receptor^23^ is regulated by a number of factors, including miRNAs^24^,^25^; nuclear factors, such as SREBP^26^, peroxisome proliferator activated receptors (PPAR)^27^ and liver X receptor (LXR)^28^,^29^. Peroxisome proliferator-activated receptors are associated with alcohol consumption^30^,^31^,^32^. Chronic alcohol intake can upregulate the hepatic expression of PPARα and PPARγ^30^. However, it has not been verified that how alcohol can effect on SR-B1 and cholesterol metabolism, and We conjecture that aboved mentioned process maybe PPAR dependent.

What is not yet clarified is how alcohol affects the blood levels of HDL-C and thus regulates cholesterol metabolism. Our aims were to determine whether (1) isolated alcohol can participate in the regulation of SR-B1 and cholesterol clearance; (2) isolated alcohol can regulate PPARγ expression; and (3) the effects of alcohol on SR-B1 and cholesterol metabolism are PPARγ dependent.

## Results

### High-Cholesterol Diet Increased the Plasma Level of Liver Enzymes and Lipids, which were Decreased through Chronic Moderate Alcohol Intake

**Figure 1:**
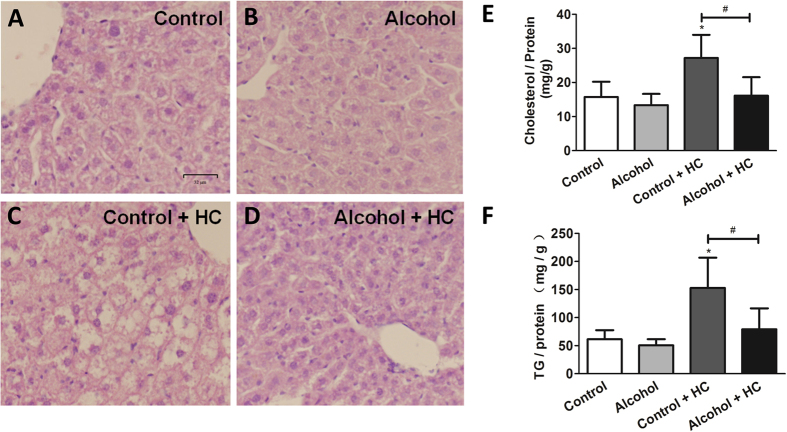
High Cholesterol Diet Increase Plasma Level of Liver Lipids Which are Decreased through Chronic Moderate Alcohol Intakes. (**A–D**) Sections of the livers from mice was subjected to H&E staining, and representative results are shown. (**E**) Total cholesterol level in liver tissues. (**F**) Total triglyceride level in liver tissues. Control, n = 8; alcohol, n = 8; control + HC, n = 8; and alcohol + HC, n = 12. Values represent means ± SD. *means P < 0.05 versus Control; ^#^means P < 0.05.

Previous reports found that long-term moderate drinking can increase blood HDL-C and reduce LDL-C^13^. In order to probe into the effects of moderate drinking on serum cholesterol and triglycerides, we assigned C57/BL6J mice into four experimental diet group: control group, alcohol group, HC group and alcohol + HC group. And then, serum lipids was investigated. it is worth noting that significant differences were found in the serum cholesterol level between the control (2.69 ± 0.21 mmol/L), control + HC (3.81 ± 0.58 mmol/L) and alcohol + HC (2.77 ± 0.68 mmol/L) groups (Table 1). TG levels were comparable among all four groups. On the other hand, we also doubt about that moderate drinking and high cholesterol diet would effect on the body health and nutritional status, we test the following index in the serum. The plasma ALB, TP andglucose levels were comparable among all four groups. LDH is an enzyme that is released during tissue damage; it is a marker of common injuries and disease. Compared with the control group (585.40 ± 108.02 U/L), the serum LDH level had no apparent changes in the alcohol group (473.00 ± 45.25 U/L) but was elevated nearly 2-fold in the HC (940.40 ± 219.07 U/L) group (Table 1). Notably, the LDH level in the alcohol + HC group (429.5 ± 112.72 U/L) decreased, similar to the level in the control group (Table 1). The serum ALT level, which is commonly measured clinically as a part of diagnostic liver function tests, was also measured. Similar to the LDH level, these levels are alike in the control and alcohol groups and obviously elevated in the HC group (159.25 ± 36.89 U/L) but returned to normal levels in the alcohol + HC group. The serum CRE and BUN levels, as monitoring indexes of renal function, were approximately the same among the four groups. Then, livers from the four dietary groups were formalin-fixed for hematoxylin and eosin staining or frozen for hepatic lipid analysis. HE staining showed that mice in the control and alcohol groups developed no apparent histological hepatic inflammation, steatosis or fibrosis during the experimental procedures (Fig. 1A,B). Mice in the HC group exerted a disorganization of the hepatocyte involving swelling and the centrilobular portion of the liver (Fig. 1C). Intriguingly, there were no appreciable hepatic histological changes in the alcohol + HC group (Fig. 1D).

Relative to the mice on the control diet, mice on the HC diet exerted a predominant increase in the hepatic triglyceride (Fig. 1F) and cholesterol (Fig. 1E) levels, while mice on the alcohol + HC diet had significantly (P < 0.05) lower hepatic lipid levels (Fig. 1E,F). Thus, chronic moderate alcohol intake is beneficial for hepatic function and can improve the abnormalities in lipid metabolism caused by a high-cholesterol diet.

### Chronic Moderate Alcohol Intakes Help Curtailing Weigh Gain caused by High Cholesterol Diet

**Figure 2:**
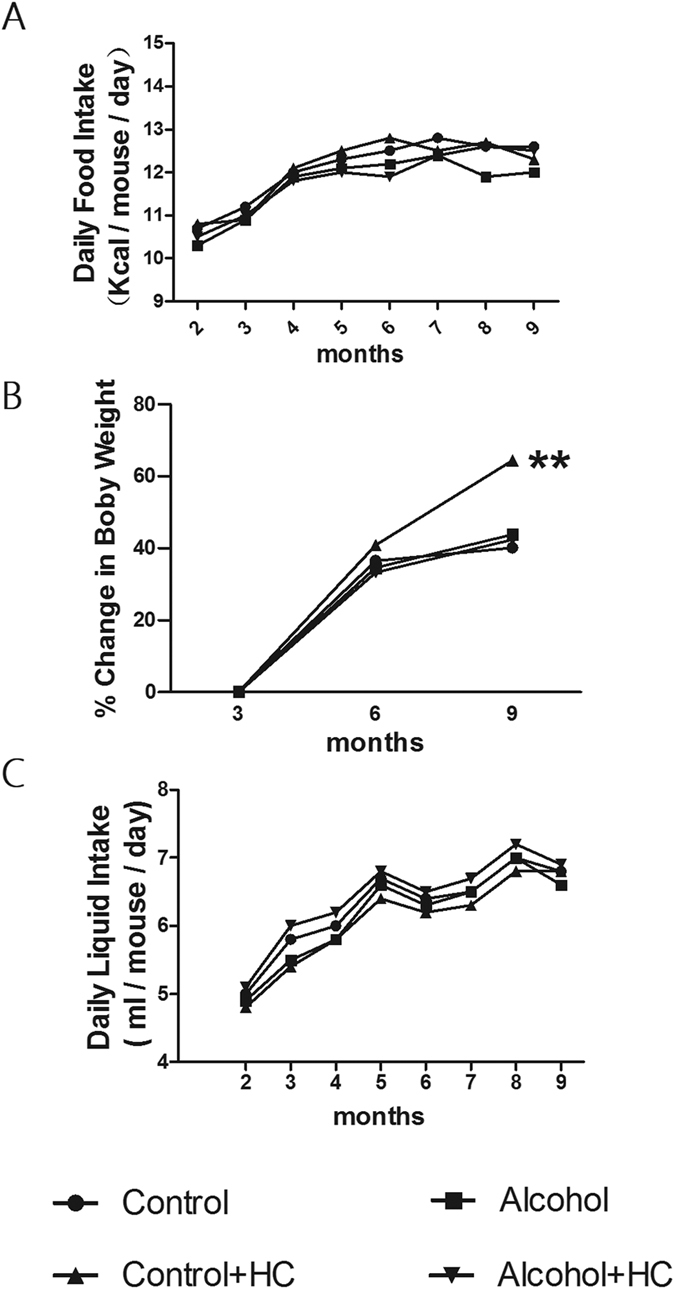
Chronic Moderate Alcohol Intakes Help Curtailing Weigh Gain caused by High Cholesterol Diet. (**A**) The average daily food intake; (**B**) Mice in the four groups had similar baseline body weight (mean 27.37 ± 0.9 g) at 3 mouth old. % of body weight increased was measured throughout experiment; (**C**) The average daily liquid (water/alcohol) intake. **means P < 0.05 versus Control.

To study dietary intake differences during the experimental procedure, daily food and liquid (water for control group and control + HC group, alcohol for alcohol group and alcohol + HC group) intake of mice was measured and shown in Fig. 2A,C. There were no obvious differences among mice in four dietary groups: daily food intake per mouse was finally stable around the level of 12 g, daily liquid intake per mouse was finally stable around the level of 6.5 ml. Intriguingly, we found changes in body weight. The four dietary groups had similar baseline weight (mean 27.37 ± 0.9 g) at 3 mouth old. % of body weight increased was measured. Body weight of mice in four groups was comparable before 6 month old.Then, mice in Control + HC group and Alcohol + HC group were fed with high cholesterol diet. At the end of the experiment, high cholesterol diet caused great body weight but alcohol + high cholesterol fed mice get no obvious weigh gain (Fig. 2B).

### Chronic Moderate Alcohol Intake Promoted Cholesterol Clearance mainly through SR-B1-Mediated Reverse Cholesterol Transport

Reverse cholesterol transport is a multi-step process resulting in the net movement of cholesterol from peripheral tissues back to the liver via the plasma^28^; it is of great significance in cholesterol metabolism. Of note, there are three important receptors on the surface of hepatocytes participating in this process: SR-B1, LRP1 and LDLR.

**Figure 3:**
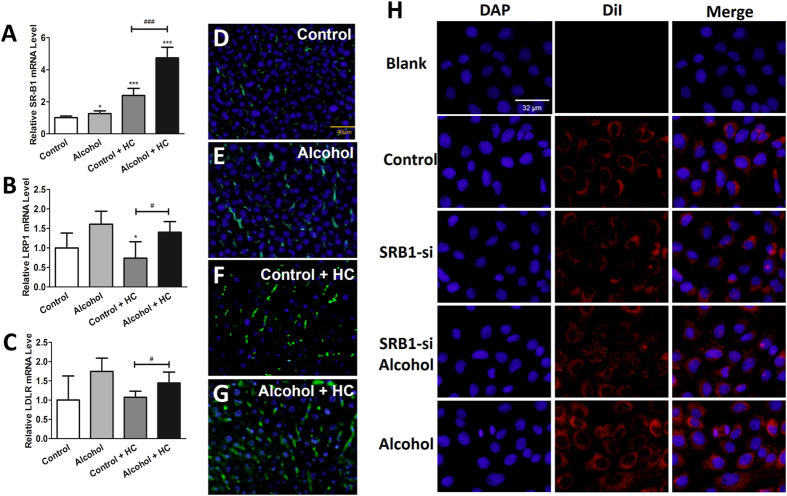
Chronic Moderate Alcohol Intakes Promote Cholesterol Clearance mainly through SR-B1 Mediated Reverse Cholesterol Transport. (**A–C**) The relative mRNA levels of SR-B1, LDLR and LRP1 were analyzed by qRT-PCR. (**D–G**) Sections of the livers were subjected to immunofluorescence staining with anti-SR-B1 (green) and DAPI (blue). (**H**) DiI-HDL uptake in AML12 cells. “Blank” indicates cells that were not incubated with DiI-HDL. Values represent means ± SD. Control, n = 8; alcohol, n = 8; control + HC, n = 8; and alcohol+ HC, n =  12. *means P < 0.05 versus Control, ***means P < 0.001 versus control; ^#^means P < 0.05.

In our study, we conjectured that alcohol promoted cholesterol clearance through accelerating reverse cholesterol transport. To test hypothesis, we analyzed the expression level of three important hepatocyte surface receptors in the liver. Compared with that of the control group, the SR-B1 mRNA level was slightly elevated (1.27-folds) in the alcohol group and (2.23-folds) in the control + HC group and most dramatically elevated (4.43-folds) in the alcohol + HC group (P < 0.05 for each comparison, Fig. 3A). LRP1 and LDLR changed only in the alcohol + HC group (Fig. 3B,C). Thus, we determined the hepatic SR-B1 protein level in four experimental groups via an immunofluorescent assay. The results showed that the protein level was consistent with the mRNA expression level (Fig. 3D–G). In an attempt to determine the effects of alcohol on selective HDL-C uptake, florescent-labeled HDL (DiI-HDL) was used. AML12 cells were transfected with SR-B1 small interfering RNA (siRNA) with or without proper ethanol treantment. Cells were harvested for mRNA analysis or an HDL-C uptake assay (Fig. 3H). Thus, alcohol participates in HDL-C uptake, and this process is mainly regulated through SR-B1-mediated reverse cholesterol transport.

### Alcohol Modulates SR-B1 Expression and Selective HDL-C Uptake through PPARγ in Mice

**Figure 4:**
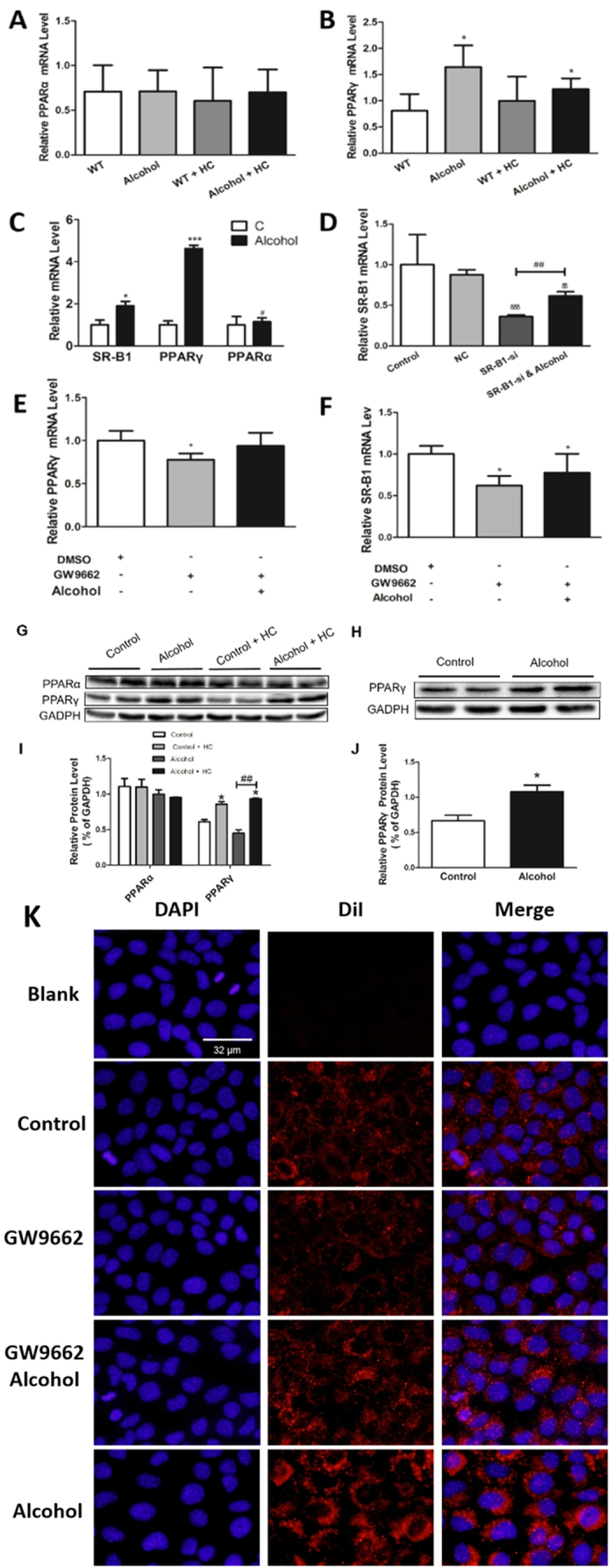
Alcohol Modulates SR-B1 Expression and Selective HDL-C Uptake through PPARγ in Mice. (**A,B**) Real time PCR analysis of the PPARα and PPARγ mRNA levels (**C**). qRT-PCR analysis of AML12 cells treated with or without alcohol for 48 h and then harvested for analysis. (**D**) AML12 cells were transfected with SR-B1 siRNA or NC and subjected to alcohol. The cells were harvested for real-time PCR analysis. (**E,F**) AML12 cells were treated with alcohol for 48 h, subjected to 1 μM GW9662 for 24 h with or without alcohol and then harvested for real-time PCR analysis. (**G,I**) The PPARα and PPARγ protein levels were analyzed and corresponding semi-quantitative analysis data was shown. (**H,J**) PPARγ protein levels were analyzed in AML12 cells and corresponding semi-quantitative analysis data was shown. (**K**) DiI-HDL uptake in AML12 cells. “Blank” indicates cells that were not incubated with DiI-HDL. Control, n = 8; alcohol, n = 8; control + HC, n = 8; and alcohol + HC, n = 12. The values represent means ± SD, *means P <  < 0.05 versus control, ***means P < 0.001 versus control; δδ means P < 0.01 versus NC; δδδ means P < 0.001 versus NC ; ^##^means P < 0.01; and ^§^means not significant.

SR-B1 is regulated by the transcription factors PPARα and PPARγ which heterodimerized with the retinoid X receptor (RXR) and bind to PPREs (peroxisome proliferator hormone response elements) regions on SR-B1^27^. To verify whether these factors are involved in the regulation of SR-B1 by alcohol, the hepatic messenger RNA analysis of PPARα/γ was performed (Fig. 4A,B). The PPARα level had no obvious differences among the four groups, while PPARγ did. Next, to gain insight into the mechanism of alcohol’s effect on reverse cholesterol transport, we treated AML12 cells with a proper concentration alcohol. We found significant (P < 0.05) elevation in the SR-B1 and PPARγ mRNA levels (Fig. 4C). SR-B1, as an admitted HDL receptor, participates in selective HDL-C uptake. The SR-B1 mRNA level was also detected after the cells were transfected with SR-B1 small interfering RNA (siRNA) and/or proper alcohol (Fig. 4D). SR-B1 expression, which was down regulated by siRNA, can be reversed by alcohol.

To further investigate the contribution of PPARγ, the PPARγ inhibitor GW9662 was used. In AML12 cells, the GW9662-treated group showed lower PPARγ and SR-B1 levels, which increased in the alcohol-treated group (Fig. 4E,F). PPARα and PPARγ were also detected by western blot, and the results are provided (Fig. 4G–J). In agreement with SR-B1 expression, HDL-C uptake exerts the same trend (Fig. 4K). In total, alcohol can modulate SR-B1 expression and selective HDL-C uptake. This process depends on PPARγ rather than on PPARα.

### Alcohol Modulated SR-B1 Expression and Selective HDL-C Uptake through PPARγ in Human Normal Hepatocytes – HL7702 Cells

**Figure 5:**
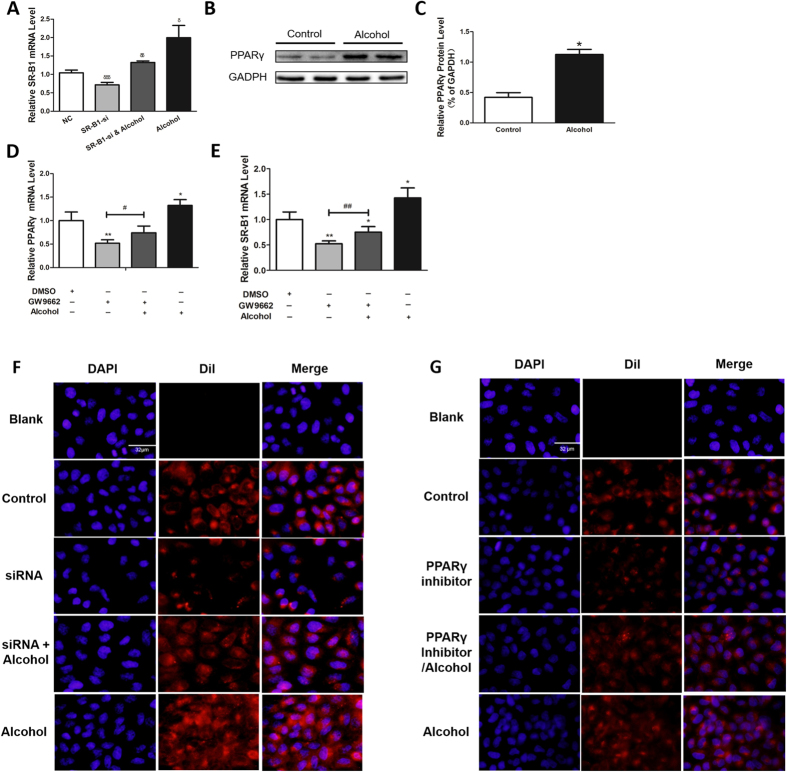
Alcohol Modulates SR-B1 Expression and Selective HDL-C Uptake through PPARγ in Human Normal Hepatocytes – HL7702 Cells. HL7702 cells were treated with alcohol for 48 h (**A**) and then harvested for real-time PCR analysis, (**B**) western blot and (**C**) corresponding semi-quantitative analysis data or (F) DiI-HDL uptake analysis. HL7702 cells were treated with alcohol for 48 h and subjected to 1 μM GW9662 for 24 h with or without alcohol, (**D,E**) and then harvested for real time PCR analysis or (**G**) DiI-HDL analysis using OLYMPUS FSX100 with a 200× objective. “Blank” indicates cells that were not incubated with DiI-HDL. Values represent means ± SD; ^δδ^means P < 0.01 versus NC; ^δδδ^means P < 0.001 versus NC; *means P < 0.05 versus control; **means P < 0.01 versus control; ^#^means P < 0.05; and ^##^means P < 0.01.

In a rare opportunity to conduct experiments in humans, we used human normal hepatocytes – HL7702 cells. In addition to the findings in AML12 cells, HL7702 cells that were treated with proper alcohol had higher SR-B1 expression level (Fig. 5A), higher PPARγ mRNA (Fig. 5D) and protein level (Fig. 5B,C) level and stronger HDL-C uptake (Fig. 5F,G). Then, HL7702 cells were transfected with SR-B1 siRNA or treated with GW9662. Finally, these cells were verified to downregulate SR-B1 expression (Fig. 5A,E) and HDL-C uptake (Fig. 5F,G). However, these effects were all reversed by alcohol (Fig. 5). These data suggest that alcohol can modulate PPARγ- and SR-B1-regulated HDL-C uptake in human cells – HL7702 cells.

### Chronic Moderate Alcohol Intake Promotes Hepatic Cholesterol Clearance by Increasing CYP7A1 Activity

**Figure 6:**
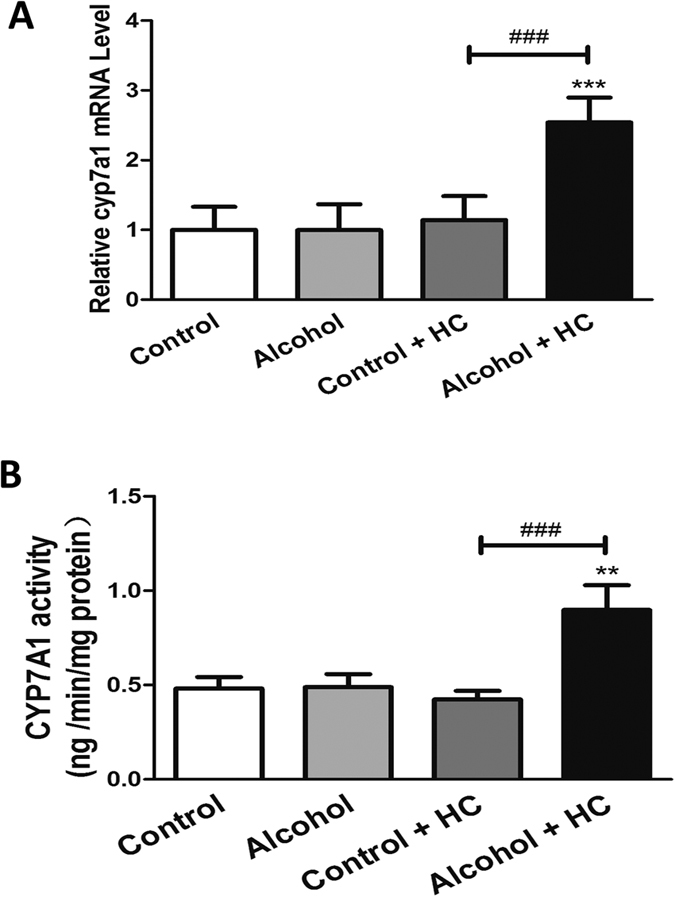
Chronic Moderate Alcohol Intakes Promote Hepatic Cholesterol Clearance through Increasing CYP7A1 Activity. (**A**) Real-time PCR analyses of the relative mRNA level of CYP7A1. (**B**) The CYP7A1 activity was measured by ELISA. Control, n = 8; alcohol, n = 8; control + HC, n = 8; and alcohol + HC, n = 12. Values represent means ± SD; **means P < 0.01 versus Control, ***means P < 0.001 versus Control; and ^###^means P < 0.001.

To further investigate how alcohol acts on cholesterol clearance, we tested the expression level of CYP7A1. CYP7A1 converts cholesterol to 7-alpha-hydroxycholesterol and it is the first and rate limiting step in bile acid synthesis. The real-time PCR analysis of mRNA levels showed a significant increase in mice that were fed a high-cholesterol and alcohol diet (Fig. 6A). The CYP7A1 activity test was in complete agreement with this result (Fig. 6B). Thus, chronic moderate alcohol intake can increase CYP7A1 activity, thereby accelerating cholesterol clearance.

## Discussion

Here, we show that the long-term administration of a small amount of alcohol can promote cholesterol clearance through a mouse model of chronic moderate drinking. Under the administration of alcohol, animals with high-cholesterol diets presented no obvious abnormalities in phenotype, blood biochemistry or hepatic lipid analysis. Our findings demonstrate the potential role and mechanism of the protective effect of alcohol and open the door for the prevention and treatment of high-cholesterol disorders.

Many epidemiological studies have reported that moderate alcohol consumption is associated with a reduced risk of cardiovascular events. St Leger A. S. *et al.* found that the French, who often drink wine, had a lower cardiovascular risk than did members of other Western societies^33^. Subsequently, alcohol was demonstrated to increase high-density lipoprotein levels and reduce low-density lipoprotein levels, platelet aggregation and the effective role of inflammation^34^. Moderate alcohol consumption can also decrease the risk of type 2 diabetes^35^. In contrast, some studies indicate the opposite effect of alcohol, finding light-to-moderate drinking to be associated with an increased risk of cancer with an established link to alcohol consumption in women^36^. However, it is very important to note that the reported benefits of alcohol have been noted predominantly in cohort epidemiological analyses to assess the effects of alcohol and wine consumption in populations. However, these studies failed to mention that white spirit, red wine and other alcoholic beverages are compound objects that cannot be used to study the effect of pure alcohol. Therefore, our mouse model of chronic moderate is drinking with pure alcohol and their drinking was autonomous which more realistically simulate human drinking habits. This model may provide a new route for research into the relationship between alcohol and diseases.

In the liver, we found that animals that were fed with high-cholesterol diets exert conspicuous steatosis and elevated triglycerides. These overall findings agree with previous results showing that SHSST protects against liver steatosis and protects vessels against inflammation arising from the excessive ingestion of cholesterol^37^. The authors of these previous studies believe that hypercholesterolemia should be considered as a risk factor for hepatic fibrosis, in addition to atherosclerosis and coronary artery disease^38^. Also shown in the study by Sohn CW was that the plasma TG level significantly increased after the feeding of high-cholesterol diets to rats^39^. Interestingly, our findings indicate no significant changes in the plasma TG but conspicuous steatosis and elevated triglycerides in the liver. This difference provides two possibilities: high-cholesterol diets have different effects among different species, or liver damage needs to reach a certain degree to cause changes in serum triglycerides.

PPARs are important transcription factors in the regulation of lipid metabolism, adipogenesis, insulin resistance and other diseases^40^. A relationship has been demonstrated between alcohol and PPARα/γ. Chronic alcohol intake can upregulate the hepatic expression of PPARα and PPARγ^30^, in addition to reduced alcohol intake in mice and the genetic association between AD or withdrawal in humans^31^. Similarly, Nakajima T found a protective role for PPARalpha in alcoholic liver disease^41^. Intriguingly, our data suggest some divergences. Liver tissues and cells mRNA were analyzed, and we found a marked variation in PPARγ but unchanged PPARα. We believe that PPARα does not participate in the process of cholesterol metabolism regulation by alcohol. It will be very interesting to see whether additional pathways participate in this process.

After the high cholesterol treatment in animal model, we found that CYP7A1 expression showed no change in HC group compared with control group. This is out of our expert. However, Peet D. J., etc. demonstrate that mice lacking the oxysterol receptor, LXR alpha, lose their ability to respond normally to dietary cholesterol and are unable to tolerate any amount of cholesterol in excess of that which they synthesize *de novo*^42^. When fed diets containing cholesterol, LXR alpha (−/−) mice fail to induce transcription of the gene encoding cholesterol 7alpha-hydroxylase. This gives us some inspirations. There is no LXR response element in the human CYP7A1 promoter sequence. So human lack of ability to respond to dietary cholesterol. We suspect that our long-term feeding may be closer to human cholesterol accumulation model. On the one hand, our result show obvious cholesterol accumulation through histopathologic and blood biochemical analysis. On the other hand, the long-term feeding way could avoid the case that LXR can not induce CYP7A1 expression in human.

In addition, we notice that the discrepancies of serum lipids and hepatic SR-B1 level between animals in the alcohol + HC group and control + HC group are more distinct compared with that between animals in the alcohol group and control group. Corroborating these notions, a high-cholesterol diet induced a state of inflammation and oxidative stress in animals^43^,^44^,^45^, in which alcohol plays a more significant role than normal diets. These data show that alcohol would exhibit a more evident protective effect in the state of inflammation and oxidative stress. Thus, it will be most interesting to evaluate the power of alcohol in the treatment of related diseases.

In summary, alcohol was found to accelerate cholesterol clearance by regulating PPARγ-dependent SR-B1-mediated reverse cholesterol transport. Concluding with theoretical and technical unknowns, subsequent studies will further characterize the other pathways and mechanisms in regulating cholesterol metabolism by alcohol and explore its role in disease treatment. These findings will provide new avenues to understand the mechanism of the protective effect by chronic moderate alcohol drinking and may achieve a new opening in therapeutic strategies for the treatment of high cholesterol-related diseases.

## Materials and Methods

### Mice and diet

Two-month-old male C57BL/6J mice were randomly assigned to one of the following four diet groups as shown in Fig. S1: (1) Control diet, standard chow diet (n = 8); (2) alcohol diet, standard chow diet with 2% (v/v) alcohol for 1 month subsequently with 3.5% (v/v) alcohol in the drinking water (n = 8) for 5 mouth plus 12 weeks; (3) Control + HC diet, 6-month standard chow diet with subsequent 12-week high-cholesterol (2% cholesterol) diet (n = 8); and alcohol + HC diet, standard chow diet with 2% (v/v) alcohol for 1 month subsequently with 3.5% (v/v) alcohol in the drinking water (n = 8) for 5 mouth and subsequent with 12-week high-cholesterol (2% cholesterol) diet with 3.5% (v/v) alcohol in the drinking water (n = 12).

Normal and high cholesterol fodder for mice were processed and purchased from keaoxieli, beijing. Mice were dept at 4–5 per cage under the conditions of 12-hour light/dark cycle and free food and water. At the end of the experiment, the mice were killed by cervical dislocation subsequent to isoflurane anesthesia. All animal experiments were approved by the Harbin Medical Univerisity’s Animal Care and Use Committee and conducted according to National Institutes of Health guidelines.

### Cell culture

Murine hepatocyte cell line AML12 cells (ATCC CRL-2254) were cultured in complete growth medium that was prepared as follows: 1:1 Dulbecco’s modified Eagle’s medium Nutrient Mixture F12 (Ham) (1:1) (Gibco, life technologies) with an insulin-transferrin-selenium mixture (Gibco, life technologies), 40 ng/mL dexamethasone (Sigma-Aldrich), 10% (v/v) fetal bovine serum (Gibco) and 50 mg/mL penicillin/streptomycin. Human normal hepatic immortal cell line HL7702 cells were grown in RPMI 1640 medium (Invitrogen) containing 10% (v/v) fetal bovine serum (Gibco). The cells were maintained in an atmosphere that was composed of 5% CO_2_ and 95% O_2_ at 37 °C.

### Cell treatment

For alcohol treatment, the cells were incubated in medium with 60 mM ethanol (Sigma-Aldrich) for 72 h. The medium was renewed every 12 hours to avoid effects caused by ethanol volatilization. PPARγ inhibitor GW9662 (Sigma-Aldrich) solution was prepared in DMSO. Cells were subjected to incubation under the following experimental conditions: (1) no treatment for 48 h, then DMSO for 24 h; (2) no treatment for 48 h and GW9662 (1 μM) for 24 h; (3) alcohol for 48 h, then alcohol and GW9662 (1 μM) simultaneously for 24 h; and (4) alcohol for 48 h, then ethanol and DMSO for 24 h.

### Gene silencing via the transfection of small interfering RNA (siRNA)

The transfection of siRNA was implemented by the application of Lipofectamine RNAiMax reagent (Invitrogen) according to the manufacturer’s instructions. SR-B1 siRNA and the scrambled negative control siRNA were obtained from GenePharma (Shanghai, China), and their working concentration and time were 100 nM for 24 h to 48 h. Then, the cells were harvested for further analysis.

### Blood chemistry

The serum of the tested mice was obtained from the supernatant of Angular venous blood after 5 min of centrifugation at 5000 rpm. Samples were then analyzed by the automatic biochemistry analyzer Sysmex Chemix-180 (Sysmex, Japan).

### Hepatic lipid analysis

The hepatic levels of triglyceride and total-cholesterol were determined using commercial kits (Applygen, Beijing, China). All of the experiments were performed according to the manufacturer’s instructions, and the results were measured using a microplate reader (Infinite 2000 PRO, TECAN, Switzerland).

### Hepatic histology

Formalin-fixed liver was dehydrated in Leica TP1020 automatic tissue hydroextractor (Leica, Germany). Then, tissues were paraffin embedded (Leica EG1150C, Leica, Germany) and sectioned (Leica SM 2000R, Leica, Germany). Liver sections were stained with hematoxylin and eosin or subjected to immunofluorescence. For immunofluorescence staining, tissue sections were deparaffinized and blocked for 40 min in 5% goat serum at room temperature. Then, they were incubated overnight at 4 °C with the primary SR-B1 antibodies (ab52629, Abcam) diluted with 2% goat serum. The sections were rinsed three times in PBST for 5 min each and incubated with anti-rabbit secondary antibodies (Life) for 1 h at RT. Then, the sections were stained with DAPI and mounted onto glass slides after washed in PBST for three times. Photomicrographs were taken using an Olympus FSX100 microscope (X200 original magnification).

### Total RNA extraction, isolation and real-time PCR analysis

The total RNA of cells and tissues was extracted with the TRIzol reagent according to the manufacturer’s instructions (Invitrogen). The quality and concentration of RNA were measured using a NanoDrop 2000c (Thermo). Only high-quality RNA samples were selected. An aliquot of 2 μg of total RNA was reverse transcribed into cDNA with a High-Capacity cDNA Reverse Transcription Kit (Applied Biosystems). SYBR Green qPCR assays were performed using Applied Biosystems 7500 Real-Time PCR System. For mouse RT-PCR analyses, the GAPDH mRNA levels were used for normalization, while β-actin was used in human cells. The primer sequences are shown in the supplemental materials.

### Western blot analysis

Tissues and cells were treated in RIPA buffer, then centrifugated at 4 °C for 15 minutes at 13500 rpm. The supernatant containing protein fraction was collected. Protein concentration was measured using the BCA protein assay kit (Applygen, Beijing, China). 50 μg of proteins were added to perform SDS—PAGE and transfered to PVDF membranes. Then menbranes were blocked with 5% skimmed milk prepared in PBST for 2 h. Primary antibodies were incubated with membranes at room temperature for 3 h and 4 °C overnight. Following are primary antibodies used: GADPH (1:4000; abcom), PPARα (1:2000; ab119416, abcom), PPARγ (1:2000; ab191407, abcom). After washing with PBST for 3 times, proteins on the membrane were probed with the appropriate horseradish peroxidase-conjugated secondary antibodies (1:2,0000; ZSBio) and chemiluminescence reagents (Haigene).

### Assays of the cellular uptake of DiI-HDL

Different cells with various treatments were under lucifuge incubation with 2 μg/ml 1,1′-dioctadecyl-3,3,3′,3′-tetramethylindocarbocyanine perchlorate (DiI)-labeled HDL (Biomedical Technologies, Stoughton, MA) for 4 h at 37 °C. For visualization, after harvesting and washing, the cells were additionally stained with DAPI and photographed using an Olympus FSX100 microscope (X200 original magnification).

### CYP7A1 activity

The CYP7A1 activity of liver tissues was detected with the Enzyme-Linked Immunosorbent Assay Kit (Cloud-Clone, Houston, USA) according to the manufacturer’s instructions.

### Statistical analysis

The results are from at least three independent experiments. GraphPad Prism version 5.01 (San Diego, CA) was used for statistical analysis. The data are presented as the mean ± SD. Student’s t-test was used for comparison values obtained from two groups. Probability values of less than 0.05 were considered statistically significant.

## Additional Information

**How to cite this article**: Li, M. *et al.* Chronic Moderate Alcohol Intakes Accelerate SR-B1 Mediated Reverse Cholesterol Transport. *Sci. Rep.* **6**, 33032; doi: 10.1038/srep33032 (2016).

## Supplementary Material

Supplementary Information

## Figures and Tables

**Table 1:** Serum parameters after 12 weeks experimental Diets.

	Control	Alcohol	Control + HC	Alcohol + HC
LDH (U/L)	585.40 ± 108.02	473.00 ± 45.25	940.40 ± 219.07*	429.50 ± 112.73^##^
AST (U/L)	87.00 ± 25.26	105.00 ± 43.84^§^	159.25 ± 36.89**	59.50 ± 9.04^## §^
ALT (U/L)	56.00 ± 28.37	29.50 ± 7.78^§^	96.00 ± 71.79	50.14 ± 13.04
ALB (g/L)	36.80 ± 2.77	36.00 ± 1.41	38.60 ± 4.88	37.29 ± 3.86
TP (g/L)	62.12 ± 4.96	64.35 ± 3.18	59.58 ± 3.94	57.03 ± 6.26
GLU (mmol/L)	7.84 ± 0.89	7.12 ± 1.15	8.58 ± 1.63	6.85 ± 1.91
CRE (μmol/L)	52.40 ± 2.94	52.90 ± 10.04	56.22 ± 6.88	56.80 ± 4.50
BUN (mmol/L)	8.57 ± 0.56	9.05 ± 0.61	8.24 ± 0.96	8.11 ± 0.63
TG (mmol/L)	1.16 ± 0.43	0.82 ± 0.01	0.93 ± 0.11	0.83 ± 0.18
T-CHO (mmol/L)	2.69 ± 0.21	2.53 ± 0.71	3.81 ± 0.58**	2.77 ± 0.68^#^
HDL-C (mmol/L)	2.24 ± 0.16	2.045 ± 0.69	3.08 ± 0.62*	2.21 ± 0.52^#^
LDL-C (mmol/L)	0.20 ± 0.02	0.24 ± 0.01	0.31 ± 0.09*	0.24 ± 0.07

Values are expressed as the mean ± SEM; Control (n = 5), Alcohol (n = 5), Control + HC (n = 5), Alcohol + HC (n = 10). *P < 0.05 vs Control ; **P < 0.01 vs Control ; ^#^P < 0.05 vs Control + HC ; ^##^P < 0.01 vs Control + HC ; ^§^means P > 0.05 vs Control.

Abbreviations: LDH, lactate dehydrogenase; AST, aspartate aminotransferase; ALT, alanine aminotransferase; ALB, albumin; TP, total protein; GLU, glucose; CRE, creatinine; BUN, blood urea nitrogen; TG, triglycerides; T-CHO, total cholesterol; HDL-C, high density lipoprotein cholesterol; LDL-C, low density lipoprotein cholesterol.
