# Supplementary material for: Chronic Moderate Alcohol Intakes Accelerate SR-B1 Mediated Reverse Cholesterol Transport
Source: Sci Rep. 2016 Sep 13;6:33032. doi: 10.1038/srep33032 (PMC5020497; doi:10.1038/srep33032)
Supplement: Supplementary Information [file srep33032-s1.doc]

Title: Chronic Moderate Alcohol Intakes Accelerate SR-B1 Mediated Reverse Cholesterol Transport

Authors: Menghua Li^1,2,#^, Yan Diao^1,2,#^, Ying Liu^3,#^, Hui Huang^1,2^, Yanze Li^1,2^, Peizhu Tan^1,2^, Huan Liang^1,2,4^, Qi He^1,2^, Junhui Nie^1,2^, Xingli Dong^1,2^, Yang Wang^1,2^, Lingyun Zhou^1,2,*^, Xu Gao^1,2,*^

^1^Department of Biochemistry and Molecular Biology, Harbin Medical University, Harbin, China. ^2^ Translational Medicine Center of Northern China, Harbin, China. ^3^Department of Gastroenterology, Heilongjiang Province Hospital, Harbin, China. ^4^Department of Clinical Laboratory, Harbin Medical University Cancer Hospital, Harbin, China.

*Corresponding author: Lingyun Zhou, Department of Biochemistry and Molecular Biology, Harbin Medical University, Harbin, China. E-mail: zhoulingyun27@sina.com or Xu Gao, Department of Biochemistry and Molecular Biology, Harbin Medical University, Harbin, China. E-mail: [gaoxu_671227@163.com](mailto:gaoxu_671227@163.com)

**Supplemental Materials**

**Fig S1.Experimental diet groups**

**
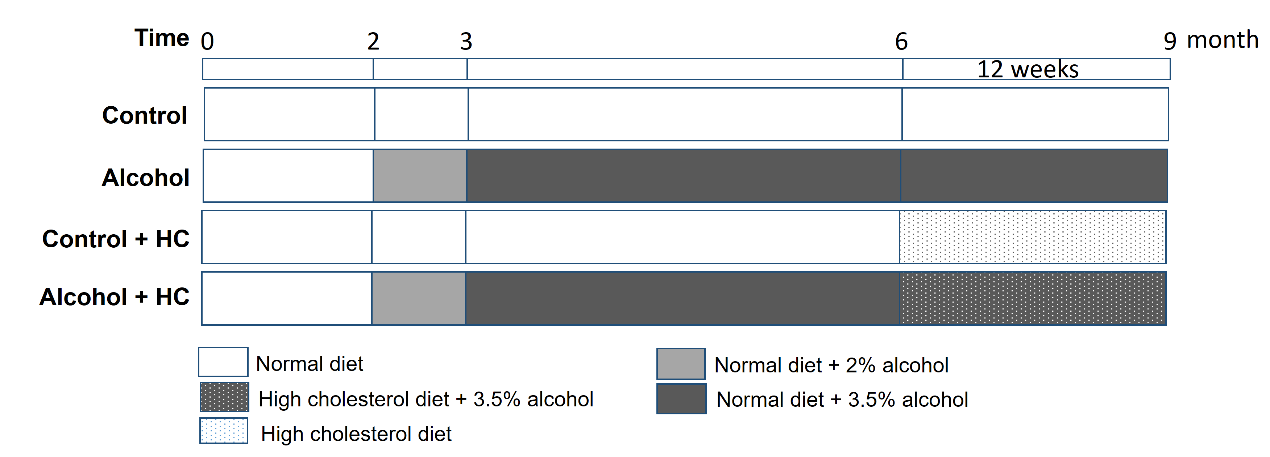
**

Figure S2

Figure S
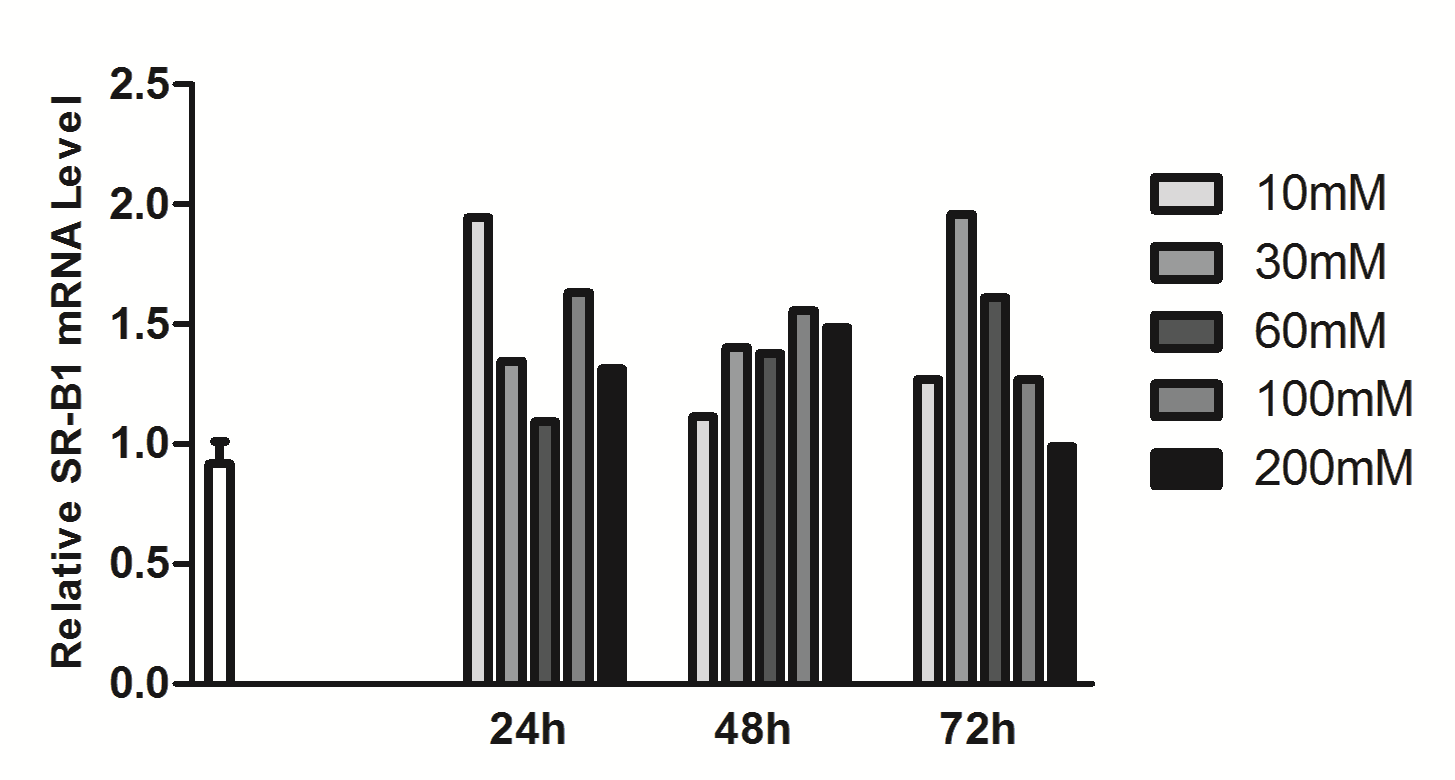
3

Figure S
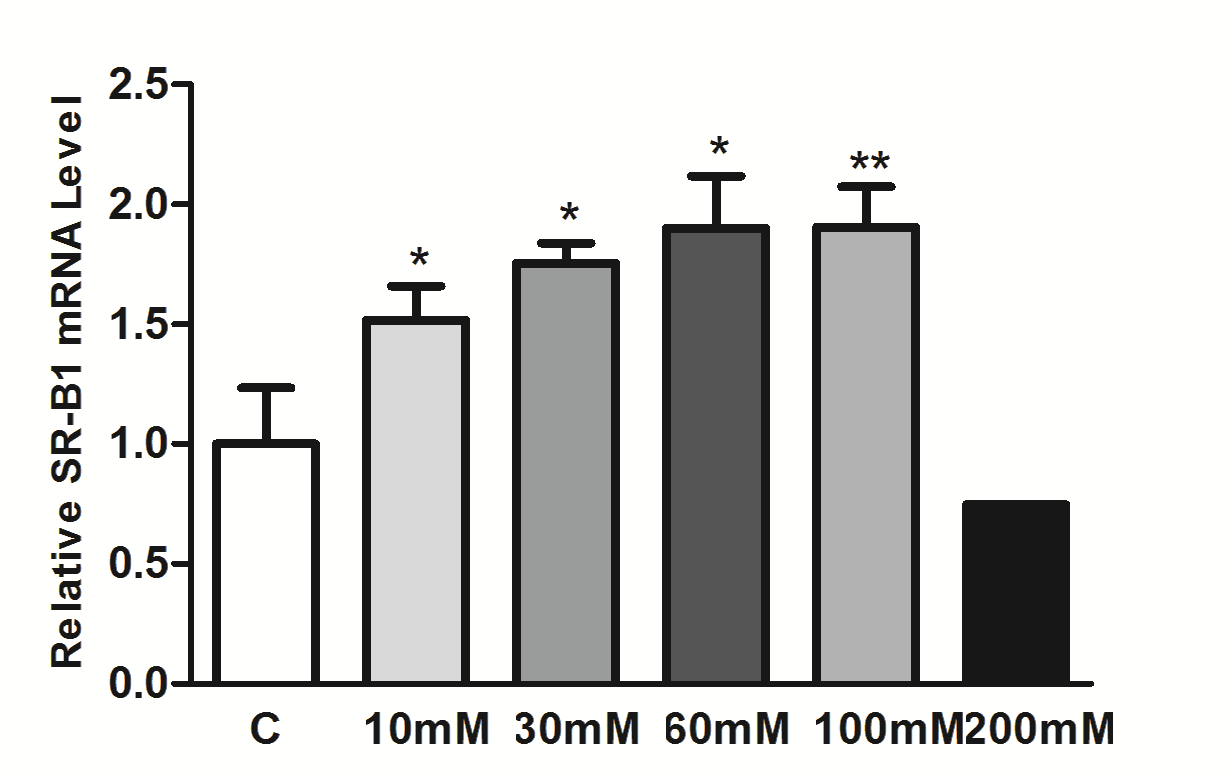
4

Figure S
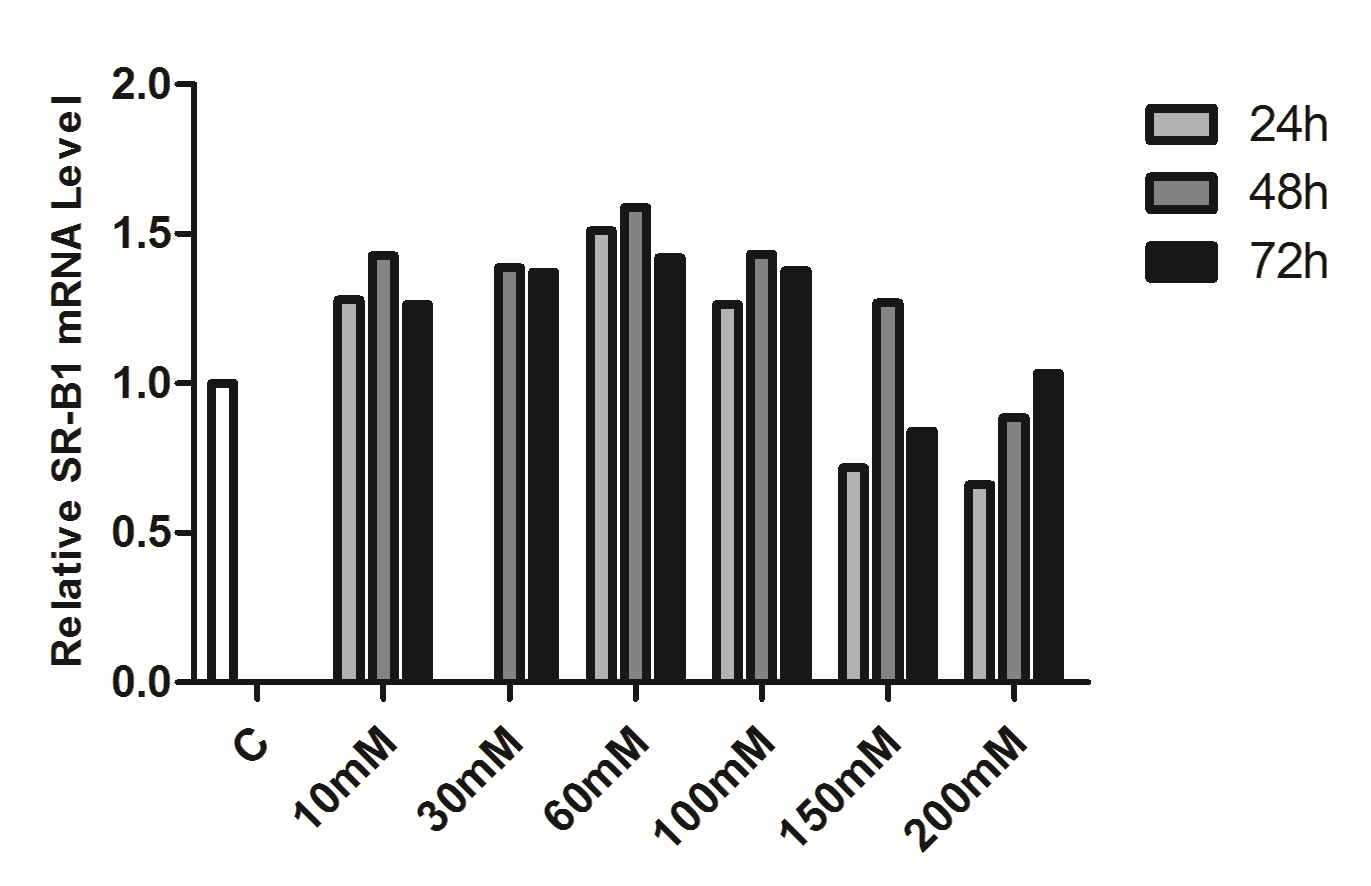
5

**
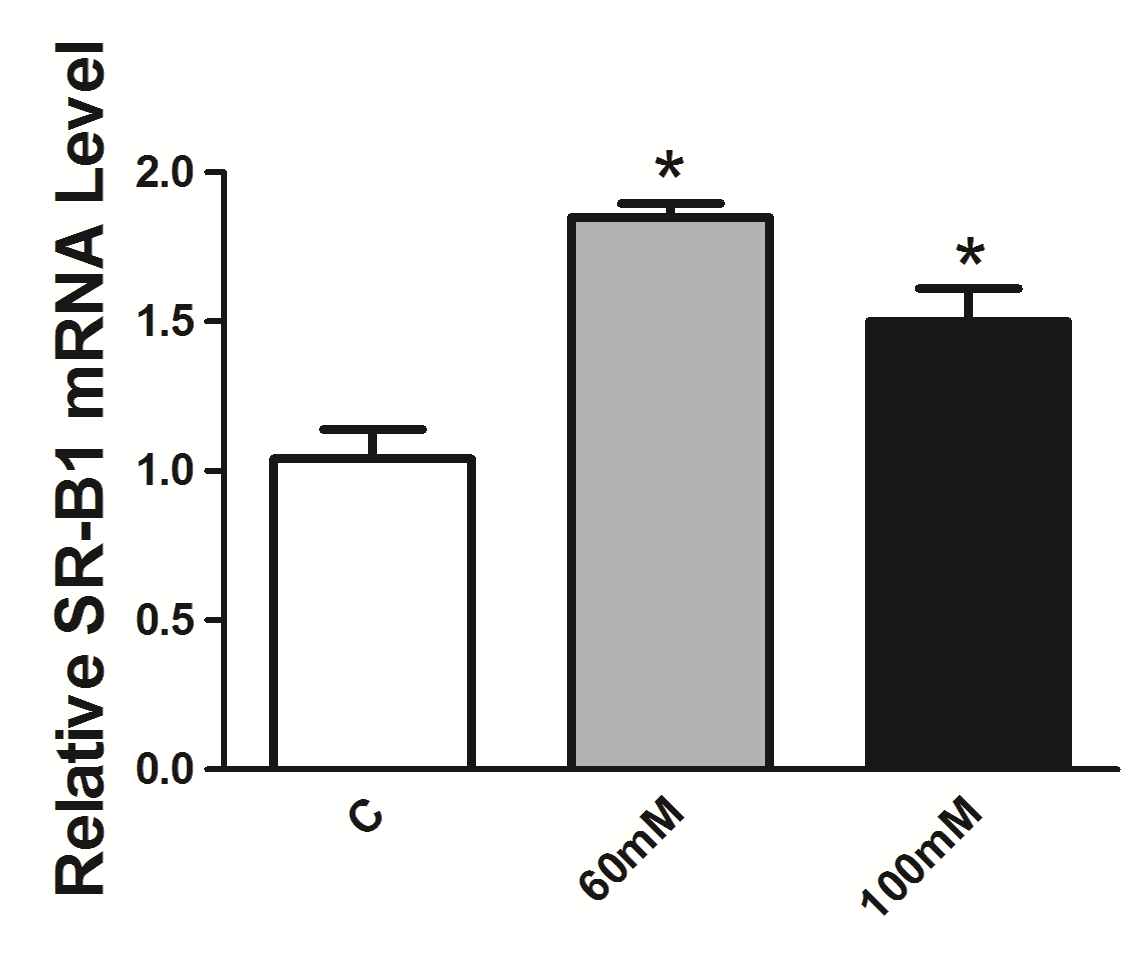
**

Figure Legends

**Fig S1.Experimental diet groups**

Figure S2. qRT-PCR analysis of AML12 cells treated with or without 0-200Mm ethanol for 0-72h, and then harvested for analysis.

Figure S3. qRT-PCR analysis of AML12 cells treated with or without 0-200Mm ethanol for 48 h, and then harvested for analysis.

Figure S4. qRT-PCR analysis of HL7702 cells treated with or without 0-200Mm ethanol for 0-72 h, and then harvested for analysis.

Figure S5. qRT-PCR analysis of HL7702 cells treated with or without 0-100Mm ethanol for 48 h, and then harvested for analysis.

**Table S1**. **Primers that were used in this article**

| **Gene** | **F** | **R** |
| --- | --- | --- |
| **GAPDH (mouse)** | GTTGTCTCCTGCGACTTCA | GGTGGTCCAGGGTTTCTTA |
| **SR-B1 (mouse)** | GCTGTATCTGGCGCTTTTTC | TCCGAATACCCTCTGGTGAG |
| **LDLR (mouse)** | TCACACAGCCTAGAGAAGTCG | ATCCTCACTGTGCTTCGGTG |
| **LRP1 (mouse)** | ACGGCTCCAACTACACACTG | TTGAGGTGCATCCTGCGAAT |
| **PPARα(mouse)** | TGGTTGAATCGTGAGGAACA | ATCGCCACTAAGGTGTCAGG |
| **PPARγ(mouse)** | ATAAAGCATCAGGCTTCCACT | GCACTTCTGAAACCGACAGTA |
| **CYP7A1 (mouse)** | TACTAGATAGCATCATCAAGGAGGCTC | CCATCCTCAAGGTGCAGAGTG |
| **HMG CoA (mouse)** | ACGATCCTTCCTTATTGGCGG | CTCCGGATCTCAATGGAGGC |
| **β-actin (human)** | TACCTCATGAAGATCCTCACC | TTTCGTGGATGCCACAGGAC |
| **SR-B1 (human)** | CATCAAGCAGCAGGTCCTTA | CGGAGAGATAGAAGGGGATAGG |
| **PPARα(human)** | TGAACAAAGACGGGATG | TCAAACTTGGGTTCCATGAT |
| **PPARγ(human)** | TCAGGGCTGCCAGTTTCG | GGGCTCCATAAAGTCACCAAAA |
